# Health screening results of Cubans settling in Texas, USA, 2010–2015: A cross-sectional analysis

**DOI:** 10.1371/journal.pmed.1003233

**Published:** 2020-08-14

**Authors:** Emma E. Seagle, Jessica Montour, Deborah Lee, Christina Phares, Emily S. Jentes

**Affiliations:** 1 Division of Global Migration and Quarantine, Centers for Disease Control and Prevention, Atlanta, Georgia, United States of America; 2 Applied Epidemiology Fellowship Program, Council of State and Territorial Epidemiologists, Atlanta, Georgia, United States of America; 3 Texas Refugee Health Program, Texas Department of State Health Services, Austin, Texas, United States of America; International Organization for Migration, SRI LANKA

## Abstract

**Background:**

Protecting the health of refugees and other migrant populations in the United States is key to ensuring successful resettlement. Therefore, to identify and address health concerns early, the US Centers for Disease Control and Prevention (CDC) recommends a domestic medical examination (screening for infectious and noninfectious diseases/conditions) shortly after arrival in the US. However, because refugee/migrant populations often have differing health patterns from one another and the US population, the collection and analysis of health information is key to developing population-specific clinical guidelines to guide the care of resettled individuals. Yet little is known regarding the health status of Cubans resettling in the US. Among the tens of thousands of Cuban migrants who have resettled in the US, some applied as refugees in Cuba, some applied for parole (a term used to indicate temporary US admission status for urgent humanitarian reasons or reasons of public benefit under US immigration law) in Cuba, and others applied for parole status after crossing the border. These groups were eligible for US government benefits to help them resettle, including a domestic medical examination. We reviewed health differences found in these examinations of those who were determined to be refugees or parolees in Cuba and those who were given parole status after arrival.

**Methods and findings:**

We conducted a retrospective cross-sectional analysis of the Texas Department of State Health Services database. Cubans who arrived from 2010 to 2015 and received a domestic medical examination in Texas were included. Those granted refugee/parolee status in Cuba were listed in federal databases for US-bound refugees/parolees; those who were paroled after arrival were not listed. Overall, 2,189 (20%) obtained either refugee or parolee status in Cuba, and 8,709 (80%) received parolee status after arrival. Approximately 62% of those who received parolee status after arrival at the border were male, compared with 49% of those who obtained prior refugee/parolee status in Cuba. Approximately one-half (45%) of those paroled after arrival were 19–34 years old (versus 26% among those who obtained refugee/parolee status in Cuba). Separate models were created for each screening indicator as the outcome, with entry route as the main exposure variable. Crude and adjusted prevalence ratios were estimated using PROC GENMOD procedures in SAS 9.4. Individuals paroled after arrival were less likely to screen positive for parasitic infections (9.6% versus 12.2%; adjusted prevalence ratio: 0.79, 0.71–0.88) and elevated blood lead levels (children ≤16 years old, 5.2% versus 12.3%; adjusted prevalence ratio: 0.42, 0.28–0.63). Limitations include potential disease misclassification, missing clinical information, and cross-sectional nature.

**Conclusions:**

Within-country variations in health status are often not examined in refugee populations, yet they are critical to understand granular health trends. Results suggests that the health profiles of Cuban Americans in Texas differed by entry route. This information could assist in developing targeted screenings and health interventions.

## Abstract 2

Emily Jentes and colleagues discuss the health of recently arrived Cuban refugees in the US.

## Author summary

**Why was this study done?:**

- Protecting the health of the tens of thousands of refugees and other migrants, including Cubans, resettling in the US is key to ensuring successful resettlement.
- Between 2010 and 2015, Cubans comprised a large portion of migrants resettling in the US arriving by one of two routes: (1) some received either refugee or parole status to enter the US while still in Cuba, and (2) some applied for parole status after arrival in the US.
- Yet little is known regarding the health status of Cubans entering the US (and, more specifically, whether the health status differed by these two routes of entry), and therefore more information is needed to inform screening procedures and health interventions for this population.

**What did the researchers do and find?:**

- We developed mathematical models using data collected from clinic visits shortly after arrival to the US that describe differences in health patterns between these two routes of entry among Cubans who arrived in the US from 2010 to 2015.
- Overall, the health patterns between the two routes of entry may differ with respect to certain health outcomes; specifically, those with parole status after arrival were less likely to screen positive for parasitic infections.
- Additionally, the results indicated that the Cuban migrant health profile is more similar to that of those born in the US compared with other migrant and refugee populations resettling in the US.

**What do these findings mean?:**

- US clinicians can use these findings to understand potential health concerns of the tens of thousands of Cubans living in the US.
- The findings can also be used to strengthen health screening guidance and can assist in developing health interventions specific to Cubans living in the US.

## Introduction

Protecting the health of refugee and other migrant populations arriving to the US is key to ensuring successful resettlement. Therefore, because these populations often have differing health patterns than those living in the US, the collection and analysis of health information is key to developing targeted clinical guidelines and interventions to assist US clinicians and public health professionals. However, little is known regarding the health status of Cubans resettling in the US. Yet in 2014, Cubans were among the top five largest groups to resettle in the US, with over 24,000 individuals resettling primarily in Florida, Texas, New York, and California [1]. From 2010 to 2015, the US witnessed an increase in the number of Cubans entering the country, and in 2015 alone, Cuban entries totaled over 43,000 individuals [2].

Historically, Cubans have entered the country as immigrants, refugees, or parolees (individuals allowed temporary admission for urgent humanitarian reasons or reasons of public benefit under US immigration law) for economic, political, and/or health reasons. A refugee is any person who is outside his or her country of nationality or habitual residence and is unable or unwilling to return to or seek protection of that country as a result of a well-founded fear of persecution based on race, religion, nationality, membership in a particular social group, or political opinion [1]. Regarding parole, Cubans could be granted such status while either still in Cuba (where a limited number of parole requests are granted through programs established by the US government, including visa lotteries and family reunification) or at the US border [2–5]. For the latter, thousands of Cubans were paroled into the US after crossing the border by boat or foot, usually via Florida or the US–Mexico border in Texas from the 1960s to 2017 [2–4]. By 2007, the predominant route for these individuals comprised travel by boat or plane to South or Central America and then over land to the US–Mexico border [6]. This route became more common given the difficulties of crossing by boat because those intercepted by the US Coast Guard prior to reaching land were returned to Cuba, as outlined in an agreement between the two countries [6]. Those who reached US soil were permitted to stay under the Cuban Adjustment Act [6]. Those granted parole status in Cuba entered through programs established by the US government, such as family reunification (2007 to present), which notified the US ahead of their arrival [2–3]. According to the Cuban Haitian Entrant Program (1980–2017) policies, all parolees regardless of entry route were eligible to apply for refugee benefits/services, including Refugee Medical Assistance (8 months of health insurance), administered by the Office of Refugee Resettlement [5]. This analysis focuses on Cuban refugees/parolees from both entry routes who received these refugee benefits/services.

Before they enter the US, immigrants, refugees, and those given parole status in Cuba undergo an overseas medical examination following the Centers for Disease Control and Prevention’s (CDC) Technical Instructions [7]. The purpose of this exam is to identify inadmissible conditions (e.g., tuberculosis disease, syphilis). If an inadmissible condition is identified during this exam, an individual would be required to undergo treatment prior to US entry. Data from these examinations are housed within CDC’s Electronic Disease Notification (EDN) system, which includes records for all refugees and Cubans who received parole status in Cuba, and are provided to states where these individuals resettle [8]. The US Department of State’s Worldwide Refugee Admissions Processing System (WRAPS), which notifies states of refugee arrivals, also includes records of Cubans granted parole status before leaving Cuba [9]. Cubans paroled at the border (i.e., upon arrival) did not receive an overseas examination and are not included in EDN records.

Regardless of entry route, Cuban refugees and parolees are recommended to undergo a voluntary domestic medical examination within 90 days of arrival in the US [5,10]. This assessment includes a comprehensive physical examination, screening for certain infectious diseases and noninfectious conditions, and vaccination updates [10]. If a positive screening is recorded, the individual is referred to appropriate care. Although the basis of the recommendations for this exam are similar across refugee populations, risk factors for certain diseases vary based on prior country of residence, age, and living circumstances. Much is known about disease prevalence among other populations resettling in the US, but the health status of Cubans has not been previously examined. Additionally, little is known regarding the differences in health status upon arrival between individuals paroled at the border and individuals who obtained refugee/parolee status in Cuba. We hypothesized that, because these two groups differed by entry route, and likely life experiences, we might also observe differences in health status. We compared health records from initial postarrival domestic medical examinations of Cubans entering Texas to identify health differences that can inform targeted screening and long-term health management strategies.

## Methods

### Participants and data collection

We conducted a retrospective cross-sectional analysis of the Texas Department of State Health Services (DSHS) database, examining differences in health status at the voluntary initial domestic (after US arrival) medical examinations among Cubans paroled at the border and those who obtained refugee/parolee status in Cuba. Cuban refugees and parolees who arrived in Texas between January 1, 2010, and September 30, 2015, and who received a domestic medical examination were included.

The Refugee Health Program within DSHS collected health screening and demographic data from all seven of the public health departments that perform domestic medical examinations for refugees and parolees. Data were retrieved from the state’s database while the program was under DSHS (in 2016 the program moved to the US Committee for Refugees and Immigrants).

DSHS data were cross-referenced with EDN and WRAPS to determine entry route [8–9]. Individuals who obtained refugee/parolee status in Cuba were identified through the presence of a record in EDN and/or WRAPS, indicating they received a predeparture overseas examination and an approval for refugee/parolee status before entry. Those who were paroled at the border were defined as individuals not listed in EDN or WRAPS. Ultimately, data analyzed only included information from the voluntary domestic medical examination for both groups. Data from the overseas examination were not included in the analysis; EDN/WRAPS data were only used to determine entry route.

Of note, all Cuban refugees/parolees listed in EDN as resettling in Texas were found in our data set (i.e., completed domestic medical examination), indicating there were no missing individuals from our denominator among those who obtained status in Cuba. Records were unavailable to determine the true denominator of those paroled at the border, but we assume not all received a domestic medical examination because it is voluntary, and therefore, there are likely some individuals not included from this group. This analysis was not guided by a specific prospective analysis plan. This study is reported as per the Strengthening the Reporting of Observational Studies in Epidemiology (STROBE) guideline (S1 STROBE Checklist).

### Demographics and health measures

Demographic data and health variables collected during the domestic medical examination included sex; age; body mass index (BMI, calculated using weight and height); blood pressure; hemoglobin and hematocrit results (to identify potential for anemia); blood lead level (BLL); laboratory screening results for hepatitis B (serologic testing of hepatitis B surface antigen, hepatitis B surface antibody, and total hepatitis B core antibody), hepatitis C (antibody test), HIV infection, and eosinophilia; and screening for *Mycobacterium tuberculosis* infection by tuberculin skin test (TST) and/or interferon-gamma release assay (IGRA). For syphilis, all clinics screened using rapid plasma reagin (RPR) followed by *Treponema pallidum* particle agglutination (TPPA) assay (three clinics also initially screened with IgG). Two clinics reported only RPR results to DSHS; all others reported results following the full sequence. Clinical results were unavailable to distinguish latent from active infection. Entrants were also screened for parasitic infections (parasites assessed at the state laboratory included *Ascaris*, *Clonorchis*, *Dientamoeba*, amoebas, *Giardia*, hookworm, and *Trichuris* by ova and parasite examination using two stool samples per individual; *Strongyloides* and *Schistosoma* by serology with an enzyme immunoassay). For all health variables, only screening results are reported. Verified clinical diagnosis data were unavailable. Table 1 provides classifications and interpretations for each health assessment component (additional information on methods for outcome measurement or cutoff values aside from that presented in the text and Table 1 was unavailable).

**Table 1:** Classification of health assessment components reported at Texas domestic medical screening examination of Cuban entrants, 2010–2015.

Health Measure	Classification	Definition
BMI^1^^,^^2^	Underweight	<18.5 kg/m^2^
	Normal	18.5–24 kg/m^2^
	Overweight	25–29 kg/m^2^
	Obesity	≥30 kg/m^2^
Blood Pressure^2^	Normal	<120 mm HG systolic and <80 mm Hg diastolic
	Elevated	≥120 mm HG systolic or ≥80 mm Hg diastolic
Potential Anemia, Based on HBG and HCT^2^^,^^3^	Positive	Abnormal reading for both HCT and HBG:
		Abnormal HBG:
		<2 years old and HBG ≤11 g/dl
		2–4 years old and HBG ≤11.1 g/dl
		5–7 years old and HBG ≤11.5 g/dl
		8–11 years old and HBG ≤11.9 g/dl
		Female 12–14 years old and HBG ≤11.8 g/dl
		Female 15–17 years old and HBG ≤12 g/dl
		Female ≥18 years old and HBG ≤12 g/dl
		Male 12–14 years old and HBG ≤12.5 g/dl
		Male 15–17 years old and HBG ≤13.3 g/dl
		Male ≥18 years old and HBG ≤13.5 g/dl
		Abnormal HCT:
		<2 years old and HCT ≤32.9%
		2–4 years old and HCT <33%
		5–7 years old and HCT ≤34.5%
		8–11 years old and HCT ≤35.4%
		Female 12–14 years old and HCT ≤35.7%
		Female 15–17 years old and HCT ≤35.9%
		Female ≥18 years old and HCT ≤35.7%
		Male 12–14 years old and HCT ≤37.3%
		Male 15–17 years old and HCT ≤39.7%
		Male ≥18 years old and HCT ≤39.9%
	Negative	Normal reading for both HBG and HCT:
		Normal HBG:
		<2 years old and HBG >11 g/dl
		2–4 years old and HBG >11.1 g/dl
		5–7 years old and HBG >11.5 g/dl
		8–11 years old and HBG >11.9 g/dl
		Female 12–14 years old and HBG >11.8 g/dl
		Female 15–17 years old and HBG >12 g/dl
		Female ≥18 years old and HBG >12 g/dl
		Male 12–14 years old and HBG >12.5 g/dl
		Male 15–17 years old and HBG >13.3 g/dl
		Male ≥18 years old and HBG >13.5 g/dl
		Normal HCT:
		<2 years old and HCT >32.9%
		2–4 years old and HCT >33%
		5–7 years old and HCT >34.5
		8–11 years old and HCT >35.4%
		Female 12–14 years old and HCT >35.7%
		Female 15–17 years old and HCT >35.9%
		Female ≥18 years old and HCT >35.7%
		Male 12–14 years old and HCT >37.3%
		Male 15–17 years old and HCT >39.7%
		Male ≥18 years old and HCT >39.9%
	Indeterminate	Abnormal HBG and normal HCT (see values above)
		Abnormal HCT and normal HBG (see values above)
BLL^2^	<5 ug/dL	<5 ug/dL (below CDC blood lead reference value)
	≥5 ug/dL	≥5 ug/dL (above CDC blood lead reference value)
Hepatitis B^2^^,^^4^^,^^5^	Infected	HBsAg+/anti-HBc+/anti-HBs−
		HBsAg+/anti-HBc−/anti-HBs−
		HBsAg+/anti-HBc+
		HBsAg+/anti-HBs+
		HBsAg+/anti-HBs−
		HBsAg+/anti-HBc−
		HBsAg+ only
	Susceptible	HBsAg−/anti-HBc−/anti-HBs−
		HBsAg−/anti-HBc−
		HBsAg−/anti-HBs−
	Immune (previous infection or vaccination)	HBsAg−/anti-HBc+/anti-HBs+ (natural infection)
		HBsAg−/anti-HBc+/anti-HBs−
		HBsAg−/anti-HBc−/anti-HBs+ (vaccination)
		HBsAg−/anti-HBc+
		HBsAg−/anti-HBs+
	Not infected, immune status unknown	HBsAg−/anti-HBc unknown/anti-HBs unknown
	Unable to interpret	HBsAg+/anti-HBc+/anti-HBs+
		HBsAg+/anti-HBc−/anti-HBs+
		Any person missing HBsAg results, regardless of other testing results
		Also in this category would be any persons with a positive HBsAg result who received the hepatitis B vaccination within 18 days before the blood draw for serologies or on the same day as but before the blood draw; however, vaccination and blood draw dates were not available to calculate this interval.
Tuberculosis Infection Screening with IGRA and/or TST^2^^,^^6^	Positive	HIV positive and TST ≥5 mm
		Age <4 and HIV negative and TST ≥10 mm
		Age ≥4 and HIV negative and TST ≥15 mm
	Negative	HIV positive and TST <5 mm
		Age <4 and HIV negative and TST <10 mm
		Age ≥4 and HIV negative and TST <15 mm
	Indeterminate	IGRA and TST listed as “indeterminate”
		IGRA or TST listed as “indeterminate” and other screening result is missing
Syphilis Screening Test^7^^,^^8^	Positive	Reported as +/− in data set
	Negative	Reported as +/− in data set
HIV^7^	Positive	Reported as +/− in data set
	Negative	Reported as +/− in data set
Hepatitis C^7^^,^^9^	Positive	Reported as +/− in data set
	Negative	Reported as +/− in data set
Parasitic Infections^7^^,^^10^	Positive	≥1 infection (each infection reported as +/− in data set)
	Negative	No infection (each infection reported as +/− in data set)
Eosinophilia^7^	Positive	Reported as +/− in data set
	Negative	Reported as +/− in data set

^1^BMI measurements from those with extreme height (<1.4 m for men and women, or >2.0 m for men and >1.9 m for women) or weight (<40 kg for men, <35 kg for women, or >136 kg for men and women) values were excluded because of possible data inaccuracies [11].

^2^CDC guidelines followed [10,12–14]; BMI and blood pressure restricted to ≥18 years old, and BLL restricted to children ≤16 years old.

^3^Assuming female is not pregnant [12].

^4^Mitruka and colleagues (2018) [15].

^5^Without complete vaccination history, limitations exist to the hepatitis B algorithm used for categorization (HBsAg−/anti-HBc+/anti-HBs− could also indicate low-level infection; HBsAg−/anti-HBc−/anti-HBs− could indicate immune from vaccination with anti-HBs waning).

^6^If both IGRA and TST were completed and recorded, the IGRA result was used in this analysis.

^7^Serologic/laboratory results were unavailable within the data set; all values were reported as positive or negative in the data set.

^8^All seven clinics screened using RPR followed by TPPA assay; three clinics also initially screened with IgG prior to this sequence; two clinics reported only RPR results (all others reported after full sequence completed).

^9^Antibody test.

^10^Parasites assessed: *Ascaris*, *Clonorchis*, *Dientamoeba*, amoebas, *Giardia*, hookworm, and *Trichuris* by ova and parasite examination using a stool sample; *Strongyloides* and *Schistosoma* by serology.

Abbreviations: anti-HBc, antibody to hepatitis B core antigen; anti-HBs, antibody to hepatitis B surface antibody; BLL, blood lead level; BMI, body mass index; CDC, Centers for Disease Control and Prevention; HBG, hemoglobin; HBsAg, hepatitis B surface antigen; HCT, hematocrit; IgG, immunoglobulin G; IGRA, interferon-gamma release assay; RPR, rapid plasma reagin; TPPA, *T*. *pallidum* particle agglutination; TST, tuberculin skin test

Because only one blood pressure reading was available (clinical guidance suggests ≥2 separate readings to diagnose), a hypertension diagnosis was not recorded; rather, individuals were classified as having “normal” or “elevated” blood pressure. Similarly, only “potential for anemia” was recorded because additional factors (pregnancy, menstruation, repeat testing) often accounted for in official diagnoses were unavailable [12]. BMI and blood pressure were analyzed for individuals ≥18 years old, and BLL for children ≤16 years, in accordance with CDC guidelines [10,13–14]. Of those screened for syphilis, 98% were ≥15 years old, as recommended by CDC [10].

### Analysis

Chi-squared comparisons were conducted to examine differences between the two entry routes (α < 0.05). Separate models were created for each component of the health assessment, with entry route as the main exposure variable. Crude and adjusted prevalence ratios (adjPRs), controlling for the county of medical examination (proxy for clinic) to limit bias due to possible differences in screening processes, were estimated using PROC GENMOD procedures (modified Poisson regression approach that ensures robust and conservative error estimation [16]; log-link function) in SAS 9.4 (Cary, NC, USA). Confounding by sex and age and two-way interactions by age (categorical variable; age as a continuous variable did not meet the linear assumption required for this type of model) with entry route and sex with entry route for each model were assessed to determine the most parsimonious model. Interaction terms were retained if significant (*P* < 0.05), and confounding terms were eliminated if the estimate did not differ by more than 10% without the inclusion of the potential confounder.

Polytomous outcomes were dichotomized for interpretability of model results. For BMI, the <2% of individuals categorized as underweight were excluded, and those with normal BMI were compared with those classified as overweight or having obesity combined. Hepatitis B was not modeled because serology results become difficult to interpret in the absence of vaccination history. Hemoglobin/hematocrit and TST/IGRA screening results classified as “indeterminate” were excluded.

### Ethical approval

This assessment was approved as nonresearch by the CDC and Texas DSHS. The Refugee Health Program of the Texas DSHS approved the use of these data for analysis. Written consent was not required.

## Results

A total of 10,898 Cubans were included. Of those, 8,709 (80%) were paroled at the border. The remaining 2,189 (20%) obtained refugee/parolee status in Cuba. Of those included, 41% were female, and the average age was 33.5 years (SD: 14.1; 13% ≤18 years old) (Table 2). Approximately 66% were overweight or had obesity (33% normal BMI, 1% underweight), and over one-half (56%) had elevated blood pressure. Four percent had a positive TST and/or IGRA, indicating further testing was required to determine tuberculosis disease (≤18 years old: 1.2%). Less than 1% of those tested screened positive for hepatitis B, and over one-half (57%) were classified as susceptible (as defined in Table 1). Ten percent screened positive for at least one parasite. In total, 130 individuals screened positive for syphilis (1.2%; all ≥18 years old), of which 92% were identified in clinics that submitted results after the full RPR/TPPA sequence. Approximately 4% had both abnormal hematocrit and hemoglobin results, suggesting potential anemia (16% abnormal hemoglobin only, 4% abnormal hematocrit only). Of the 1,178 children with valid BLL results, 8% recorded a level higher than CDC’s reference level of ≥5 ug/dL (0.8% ≥10 ug/dL).

**Table 2:** Demographic characteristics and clinical screening results of individuals from Cuba resettling in Texas from 2010 to 2015 by route of entry (*N*, %).

Characteristic	All	Paroled into US at Border (*N* = 8,709)^1^	Obtained Refugee/Parolee Status in Cuba (*N* = 2,189)^1^	*P* Value^2^
	(*N* = 10,898)^1^			
Female	4,464 (41.0)	3,346 (38.4)	1,118 (51.1)	<0.001
Age				
<19 years old	1,443 (13.2)	836 (9.6)	607 (27.7)	<0.001
19–34 years old	4,508 (41.4)	3,944 (45.3)	564 (25.8)	
35–44 years old	2,715 (24.9)	2,237 (25.7)	478 (21.8)	
≥45 years old	2,232 (20.5)	1,692 (19.4)	540 (24.7)	
BMI^3^				
Underweight	129 (1.4)	92 (1.2)	37 (2.5)	<0.001
Normal	2,995 (33.0)	2,476 (32.7)	519 (34.4)	
Overweight	3,815 (42.0)	3,242 (42.8)	573 (38.9)	
Obesity	2,151 (23.7)	1,770 (23.4)	381 (25.2)	
Blood Pressure^3^				
Normal	3,307 (30.4)	2,764 (31.7)	543 (24.8)	0.515
Elevated	6,102 (56.0)	5,068 (58.2)	1,034 (47.3)	
Hepatitis B				
Infected	71 (0.7)	54 (0.6)	17 (0.8)	0.020
Susceptible	6,237 (57.2)	4,927 (56.6)	1,310 (59.8)	
Immune (prior infection or vaccination)	4,445 (40.8)	3,615 (41.5)	830 (37.9)	
Hepatitis C				
Positive	18 (0.2)	9 (0.1)	9 (0.4)	
Negative	2,586 (23.7)	1,766 (20.3)	820 (37.5)	0.097
Tuberculosis Infection (IGRA or TST)^4^				
Positive	377 (3.5)	304 (3.5)	73 (3.4)	0.689
Negative	10,319 (95.9)	8,234 (95.8)	2,085 (96.3)	
Indeterminate	61 (0.6)	54 (0.6)	7 (0.3)	
Syphilis Screening Test (RPR/TPPA)^5^				
Positive	130 (1.2)	111 (1.3)	19 (0.9)	0.286
Negative	9,823 (90.1)	8,030 (92.2)	1,793 (81.9)	
HIV				
Positive	58 (0.5)	53 (0.6)	5 (0.2)	
Negative	10,695 (98.1)	8,582 (98.5)	2,113 (96.5)	0.033
Parasitic Infection(s)				
≥1 parasite detected	1,098 (10.1)^6^^,^^7^	834 (9.6)	264 (12.1)	
No parasites detected	9,800 (89.9)	7,875 (90.4)	1,925 (87.9)	0.001
Eosinophilia				
Positive	916 (8.4)	648 (7.4)	268 (12.2)	<0.001
Negative	9,801 (89.9)	7,933 (91.1)	1,868 (85.3)	
Potential Anemia				
HBG and HCT normal	9,056 (83.1)	7,304 (83.9)	1,752 (80.0)	0.015
HBG and HCT abnormal	409 (3.8)	308 (3.5)	101 (4.6)	
Indeterminate: HBG normal, HCT abnormal	31 (0.3)	16 (0.2)	15 (0.7)	
Indeterminate: HCT normal, HBG abnormal	1,294 (11.9)	997 (11.5)	297 (13.6)	
Blood Lead Level^3^^,^^8^				
≥5 ug/dL	97 (8.2)	35 (5.2)	62 (12.3)	<0.001
<5 ug/dL	1,081 (91.8)	638 (94.8)	443 (87.7)	

^1^Where percentages do not add to 100%, values were missing in the data set.

^2^Chi-squared statistics; indeterminate results were excluded from chi-squared analysis.

^3^Centers for Disease Control and Prevention guidelines followed [10,12–14]; BMI and blood pressure restricted to ≥18 years old, normal blood pressure (<120 mm HG systolic and <80 mm Hg diastolic), elevated blood pressure (≥120 mm HG systolic or ≥80 mm Hg diastolic), blood lead levels restricted to children ≤16 years.

^4^If both IGRA and TST were completed and recorded, the IGRA result was used in this analysis.

^5^All seven clinics screened using RPR followed by TPPA assay; three clinics also initially screened with IgG prior to this sequence; two clinics reported only RPR results (n = 11 of these screened positive; all others reported after full sequence completed).

^6^*Ascaris*, 0.1%; *Clonorchis*, 0%; *Dientamoeba*, 0.5%; amoebas, 5.3%; *Giardia*, 2.2%; hookworm, 0.2%; *Trichuris*, 0.2%; *Strongyloides*, 2.2%; *Schistosoma*, 0.02%.

^7^9.5% positive for only one, 0.5% positive for two, and 0.1% positive for three.

^8^Ten individuals (0.8%) had a blood lead level value of ≥10 ug/dL.

Abbreviations: BMI, body mass index; HBG, hemoglobin; HCT, hematocrit; IgG, immunoglobulin G; IGRA, interferon-gamma release assays; RPR, rapid plasma reagin; TPPA, *T*. *pallidum* particle agglutination; TST, tuberculin skin test.

**Fig 1:**
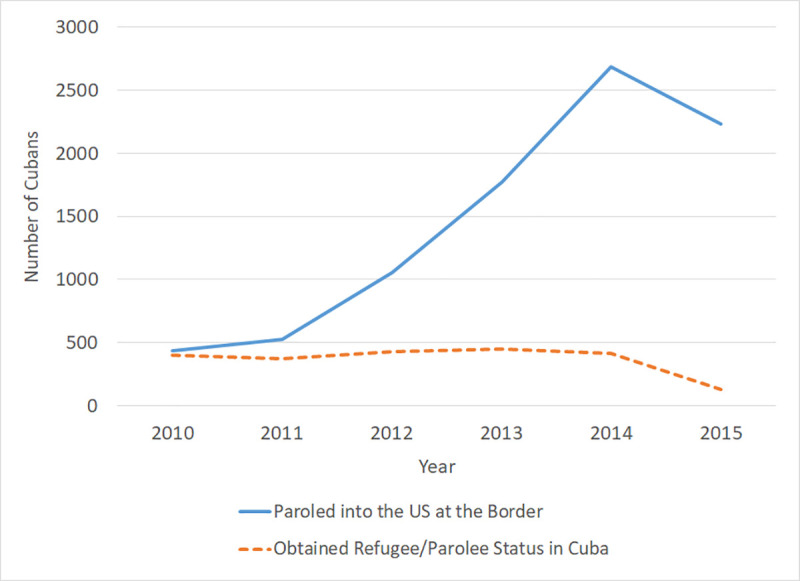
Number of Cubans entering the US and settling in Texas during 2010–2015 by entry route.

Of those included in the analytic cohort, the majority of individuals paroled at the border entered after 2012 (solid line, Fig 1), and the number of individuals who obtained refugee/parolee status in Cuba remained relatively unchanged between years (Fig 1). Approximately 62% of those paroled at the border were male, compared with 49% who obtained status in Cuba. Approximately half (45%) of those paroled at the border were 19–34 years old (versus 26% among those who received status in Cuba). Chi-squared tests revealed significant differences in gender, age, BMI, hepatitis B infection and susceptibility, HIV infection, parasitic infection(s), presence of eosinophilia, potential anemia, and elevated BLL (EBBL) between the two entry routes (Table 2).

Table 3 presents crude prevalence ratios and adjPRs. Females paroled at the border were more likely than females who received status in Cuba to be overweight or have obesity (adjPR: 1.06 [1.02–1.11]; controlling for age). This correlation was the opposite for males (adjPR: 0.88 [0.85–0.92]; controlling for age). For both sexes, those paroled at the border were less likely to be infected with at least one parasite (adjPR: 0.79 [0.71–0.88]) but more likely to have eosinophilia (females adjPR: 2.00 [1.80–2.23], males adjPR: 1.48 [1.26–1.73]). Males paroled at the border were more likely to have a positive syphilis screening (adjPR: 1.24 [1.06–1.46]), whereas their female counterparts were less likely (adjPR: 0.32 [0.12–0.80]). Among the 1,178 children ≤16 years old, those paroled at the border were less likely to have EBLL of ≥5 ug/dL (adjPR: 0.42 [0.28–0.63]).

**Table 3:** Crude and adjusted PR of domestic medical screening status, Cubans in Texas who were paroled into the US at the border versus those who obtained refugee/Cuban entrant status in Cuba (reference), 2010–2015.

Characteristic	*N* for Crude Model	Crude PR (95% CI)^1^	*P* Value	*N* for Adjusted Model	Adjusted PR (95% CI)	*P* Value	Interaction and confounder terms included in each model^2^
BMI^3^							
Normal	8,961	ref	0.0086	8,961	ref	Females: 0.0949	age, sex, age*border, sex*border, county
Overweight or obese		1.03 (1.01–1.06)			Females^9^: 1.04 (0.99–1.08)	Males: <0.001	
					Males^9^: 0.88 (0.85–0.91)		
Blood Pressure^3^							
Normal	9,409	ref	0.2085	9,409	ref	Females: <0.001	age, sex, age*border, sex*border, county
Elevated		0.99 (0.97–1.01)			Females^10^: 1.08 (1.04–1.12)	Males: 0.2624	
					Males^10^: 0.98 (0.95–1.01)		
Eosinophilia							
Negative	10,717	ref	<0.001	10,717	ref	Females: <0.001	age, sex, sex*border, age*border, county
Positive		0.60 (0.55–0.66)			Females^11^: 1.78 (1.55–2.04)	Males: 0.0046	
					Males^11^: 1.23 (1.07–1.43)		
Hepatitis C							
Negative	2,604	ref	0.0203	2,604	ref	0.0021	age, county
Positive		0.47 (0.25–0.89)			0.41 (0.23–0.72)		
Tuberculosis Infection (IGRA or TST)^4^^,^^5^							
Negative	10,510	ref	0.7946	10,510	ref	Females: 0.4677	sex, sex*border, county
Positive		1.05 (0.72–1.55)			Females: 1.18 (0.75–1.86)	Males: 0.4269	
					Males: 0.85 (0.57–1.27)		
Syphilis Screening Test (RPR/TPPA)^6^							
Negative	9,953	ref	0.0229	9,938	ref	Females: 0.8036	sex, sex*border, county
Positive		1.30 (1.04–1.63)			Females: 0.32 (0.12–0.80)	Males: 0.0090	
					Males: 1.24 (1.06–1.46)		
HIV							
Negative	10,753	ref	<0.001	10,390	ref	0.0045	sex, county
Positive		2.60 (1.53–4.43)			2.21 (1.28–3.82)		
Parasitic Infection(s)							
No parasites detected	10,898	ref	<0.001	10,898	ref	<0.001	county
≥1 parasite detected		0.79 (0.71–0.88)			0.79 (0.71–0.88)		
Potential Anemia^5^^,^^7^^,^^8^							
Both (HBG and HCT) normal	10,759	ref	0.0367	10,759	ref	0.1985	sex, county
Both (HBG and HCT) abnormal		0.76 (0.59–0.98)			0.83 (0.64–1.08)		
Blood Lead Level^3^							
≥5 ug/dL	1,178	ref	<0.001	1,609	ref	<0.001	none
<5 ug/dL		0.42 (0.28–0.63)			0.42 (0.28–0.63)		

^1^County was included in the crude model to control for potential bias due to unknown differences between clinics in screening procedures.

^2^An asterisk (*) denotes interaction between the two terms; terms without an asterisk are confounders.

^3^Centers for Disease Control and Prevention Guidelines followed [10,12–14]; BMI and blood pressure restricted to ≥18 years old, blood lead levels restricted to children ≤16.

^4^If both IGRA and TST were completed and recorded, the IGRA result was used in this analysis.

^5^“Indeterminate” classifications were excluded from models.

^6^All seven clinics screened using RPR followed by TPPA assay; three clinics also initially screened with IgG prior to this sequence; two clinics reported only RPR results (all others reported after full sequence completed)

^7^Using HBG and HCT results.

^8^Sensitivity analysis that categorizes potential anemia as abnormal HBG or abnormal HCT: 16% within anemia in total population (15% among those paroled at the border, 19% among those who obtained status in Cuba); crude PR: 0.80 (0.75–0.86); adjusted PR: 0.94 (0.87–1.01).

^9^Females: 19–34 years old PR = 0.82 (0.72–0.93), 35–44 years old PR = 0.99 (0.94–1.04), >44 years old PR = 0.89 (0.82–0.98); males: 19–34 years old PR = 1.02 (0.95–1.10), 35–44 years old PR = 1.07 (0.96–1.18), >44 years old PR = 0.94 (0.90–0.99).

^10^Females: 19–34 years old PR = 0.97 (0.91–1.04), 35–44 years old PR = 0.98 (0.93–1.04), >44 years old PR = 1.11 (1.06–1.15); males: 19–34 years old PR = 1.04 (0.98–1.09), 35–44 years old PR = 1.01 (0.97–1.05), >44 years old PR = 1.01 (0.97–1.06).

^11^Females: 0–18 years old PR = 1.82 (1.38–2.40), 19–34 years old PR = 1.20 (0.77–1.88), >35–44 years old = 1.74 (1.03–2.95), >44 years old PR = 2.46 (1.73–3.49); males: 0–18 years old PR = 1.31 (1.20–1.43), 19–34 years old PR = 1.02 (0.79–1.31), 35–44 years old PR = 1.19 (0.91–1.56), >44 years old PR = 1.63 (1.31–2.02).

Abbreviations: BMI, body mass index; CI, confidence interval; HBG, hemoglobin; HCT, hematocrit; IgG, immunoglobulin G; IGRA, interferon-gamma release assay; PR, prevalence ratio; ref, reference; RPR, rapid plasma regain; TPPA, *T*. *pallidum* particle agglutination; TST, tuberculin skin test.

## Discussion

Overall, our analysis suggests that the health profiles of individuals paroled into the US at the border and those who obtained refugee/parolee status in Cuba during 2010–2015 may differ with respect to certain health outcomes (e.g., BLLs in children). However, for some health outcomes, our results are inconclusive. The mechanisms for the patterns we observed remain unclear but may include differences in exposures both en route and before departure (potentially related to socioeconomic status or education); duration of the journey from Cuba to the US, which potentially impacted presentation or resolution of the health condition; and receipt of overseas medical examination and treatments provided. Additionally, human migration is complex and influenced by a variety of geopolitical and socioeconomic factors. Therefore, it is also possible the differences observed occurred by chance or were driven by self-selection of route.

Surprisingly, we observed a higher risk for EBLL among children who obtained refugee/parolee status in Cuba. Such patterns could be attributed to differences in exposures within Cuba associated with residential locations, family members’ occupations, or unknown socioeconomic factors. For instance, prior analyses found high prevalence of EBLL among Cuban refugee/immigrant children associated with car battery exposure and/or contact with adults whose occupations involved car repair in Cuba [17]. Yet any potential association with these factors and entry route remains unclear. Additionally, although Cuba restricted lead paint use in 1934 [18], the allowable level of lead in paint remains high [19], and the use of leaded gasoline was not restricted until after 1998 [20]. Given the potential for lead persistence in the environment [21], individuals of both entry routes were potentially exposed in Cuba. However, presentation with elevated lead levels in the US could depend on time since exposure. For instance, little is known regarding average time spent en route for those paroled at the border; however, it is estimated that some resided in South and/or Central America for months or years [6]. Those who received refugee/parolee status in Cuba typically embarked on direct flights, spending minimal time in travel [6]. Therefore, given our understanding of migration patterns from Cuba, time between last lead exposure in Cuba and the US medical examination was likely greater among those paroled at the border, providing a larger window for elevated levels to decline [22]. Additional analyses are needed to understand the impacts of length of journey and exposures within Cuba on BLL among Cubans in the US.

Syphilis screening positivity was slightly higher among those paroled at the border (1.3% versus 0.9% among those who received status in Cuba). Additionally, our results suggest a higher prevalence of infection (latent or active) among males paroled at the border. However, the RPR/TPPA testing sequence serves only as a screening and cannot provide a confirmed diagnosis of current infection or determine whether an individual is infectious due to testing limitations (i.e., tests are not specific to syphilis, can be impacted by conditions such as pregnancy or immune disorders, and cannot distinguish prior infection from active disease) [23]. A definitive diagnosis requires a medical history and physical examination, which our analysis does not include [23]. Therefore, our values may overestimate active infections because of the potential inclusion of those with previous syphilis (resolved or treated) or those currently or previously positive for other treponemal infections. We were unable to obtain medical or treatment history and thus could not calculate the degree of overestimation. Additionally, comparisons to the US population are difficult because our analysis estimated prevalence, whereas syphilis is typically measured using incidence rates of reported primary and secondary syphilis cases (2016: 8.7 per 100,000 [24]), and no comparable US analyses have examined only RPR/TPPA positivity. Even US-based prevalent syphilis infections estimates (117,000 estimated infected in 2008) are not entirely comparable, given the likely inclusion of previous infections in our estimates [25]. Ultimately, syphilis is treated during the overseas medical examination, possibly contributing to the lower overall prevalence among those who received refugee/parolee status in Cuba, yet reasons for differences by sex remain unknown. [7,10].

We expected a higher prevalence of parasitic infections among those paroled at the border given their journey through regions that potentially lacked adequate sanitation and have higher parasitic infection rates compared with Cuba [26–27]. However, our analysis indicated that those who obtained refugee/parolee status in Cuba were more likely to screen positive for parasites. On the other hand, those who crossed the border were more likely to have eosinophilia, often considered a nonspecific proxy for parasitic infections. These discrepant results could be due to other undiagnosed parasitic infections, yet additional data are needed to disentangle this association.

In addition to understanding how these two groups compare, it is also important to compare with the US and other refugee populations. Using this information, clinicians can adjust their screening and intervention strategies to reflect the population being served. For example, we found that combined overweight and obesity prevalence was similar between the adult US population and the adult Cuban population (66% Cubans versus 71% US population; comparison limited by differences in age distributions) [28]. BLLs in children from Cuba were higher than US levels [29]. Compared with many other refugee populations, tuberculosis and hepatitis B infection prevalences were lower among Cubans [15,30]. In general, the Cuban refugee/parolee health profile is more similar to the US population than that of many other refugee populations.

Our analysis was subject to several limitations. Only screening results were used, indicating potential for misclassification and/or prevalence overestimation/underestimation in the event of indeterminate or false positive/negative results. Secondly, only 2010–2015 Texas data were used, and therefore, the results may not be representative of Cubans who resettled outside of Texas or in recent years. Entry route misclassification also cannot be ruled out (e.g., individual received an overseas medical examination but chose to cross the border). Although suspected to be small, it is unclear what proportion could have been misclassified in this manner. Only those who received refugee/parolee status in Cuba received an overseas medical exam; however, because this exam only screens for inadmissible conditions, the impact would only occur in the syphilis and tuberculosis models. Nonetheless, our ultimate goal is to understand health differences between the two groups upon arrival to the US to appropriately treat and care for them after arrival. Therefore, although we cannot control for the medical care prior to US arrival, we do not believe this to be of major issue in terms of our overall message. Additionally, the data set is likely missing individuals paroled at the border who did not receive a domestic medical screening. This omission potentially introduced bias if these individuals were demographically or clinically different from those examined; however, data are not available to assess whether differences exist. Lack of access to clinic-level information prevented the use of laboratory-specific cutoff values for abnormal results, and missing information limited our ability to make complex inferences (e.g., information about pregnancy, which impacts hemoglobin/hematocrit results interpretation, and history of Bacille Calmette–Guerin vaccine, which may cause a false positive TST reaction, were unavailable) [31]. Additionally, without vaccination history, hepatitis B serologic testing interpretation is difficult because, for some, hepatitis B surface antibody wanes postvaccination, yet protection persists through immune memory. In other cases, differentiation between susceptibility and low-level hepatitis B infection using only serology results is difficult. Although 9 of the 10 models included >80% of the study population, missing data limited our interpretations, particularly for hepatitis C. Lack of data availability on socioeconomic status or education level also prevented the ability to adjust our models based on these factors. Ultimately, our estimates are merely a cross-sectional view and can neither identify a directional relationship nor account for differences related to travel time or geographic route. Finally, given the large sample size, even minimal differences were significant. Yet differences in lead levels, parasitic infections/eosinophilia, and syphilis have clinical and public health significance and should continue to be investigated.

Although the Cuban Haitian Entrant Program’s policies changed in 2017 regarding those paroled at the border [5], thousands of Cubans entered under its premises [2,6,32]. As outlined, among Cubans residing in the US, there exist two distinct subpopulations that differ in not only life experiences and entry routes but also health profiles. Therefore, understanding the health status of these two groups can be used to inform US-based public health recommendations and develop intervention strategies targeted to each subpopulation.

### New contribution to literature

Few analyses have been conducted among Cuban entrants, with most focusing on policy changes across time. Only a small number focused on health, as does our analysis. Additionally, within-country variations in health status are not often examined in refugee populations, yet they are critical to understand granular health trends. Our analysis provides a more in-depth view of the health profiles among Cubans upon US arrival and suggests that, although they shared a common country of origin, the health profiles of those paroled at the US border and those who obtained refugee/parolee status in Cuba were different.

## Supporting information

S1 STROBE Checklist STROBE, strengthening the reporting of observational studies in epidemiology. (DOCX)

## Abbreviations

adjPR: adjusted prevalence ratio
BLL: blood lead level
BMI: body mass index
CDC: Centers for Disease Control and Prevention
DSHS: Department of State Health Services
EBBL: elevated BLL
EDN: Electronic Disease Notification
IGRA: interferon-gamma release assay
RPR: rapid plasma reagin
STROBE: Strengthening the Reporting of Observational Studies in Epidemiology
TPPA: *Treponema pallidum* particle agglutination
TST: tuberculin skin test
WRAPS: Worldwide Refugee Admissions Processing System

## References

[1] Office of Refugee Resettlement. Fiscal year 2014 refugee arrivals Washington: Office of Refugee Resettlement; 2015 2 11 [cited 2018 Jun 1]. Available from: https://www.acf.hhs.gov/orr/resource/fiscal-year-2014-refugee-arrivals.

[2] Batalova J, Zong J. Cuban immigrants in the United States. Washington: Migration Policy Institute; 2020 6 11 [cited 2018 Jun 14]. Available from: https://www.migrationpolicy.org/article/cuban-immigrants-united-states#Distribution.

[3] Florida Department of Children and Families. Refugee program eligibility guide for service providers. Florida Department of Children and Families; [cited 2018 Jun 2]. Available from: https://www.myflfamilies.com/service-programs/refugee-services/overview.shtml.

[4] Luna K. Growing numbers of Cuban migrants in the United States. Washington: Center for Immigration Studies; 2016 5 7 [cited 2018 Jun 4]. Available from: https://cis.org/Report/Growing-Numbers-Cuban-Migrants-United-States.

[5] US Citizenship and Immigration Services. Cuban Haitian Entrant Program (CHEP). Washington: US Citizenship and Immigration Services; [cited 2018 Jun 27]. Available from: https://www.uscis.gov/archive/cuban-haitian-entrant-program-chep.

[6] Wasem RE. Cuban migration to the United States: policy and trends. Washington: Congressional Research Service; [cited 2018 Aug 4]. Available from: https://fas.org/sgp/crs/row/R40566.pdf.

[7] Centers for Disease Control and Prevention. Technical Instructions for Panel Physicians and Civil Surgeons. Atlanta: CDC; 2016 11 23 [cited 2018 Jun 1]. Available from: https://www.cdc.gov/immigrantrefugeehealth/exams/ti/index.html.

[8] Lee D, Philen R, Wang Z, McSpadden P, Posey DL, Ortega LS, et al Disease surveillance among newly arriving refugees and immigrants—Electronic Disease Notification System, United States, 2009. MMWR Surveill Summ. 2013;62(7):1–20. 24225411

[9] wrapsnet.org [Internet]. Worldwide refugee admissions processing system. Arlington, VA: Refugee Processing Center; 2016 [cited 2018 Jun 2]. Available from: http://www.wrapsnet.org/.

[10] Centers for Disease Control and Prevention. Guidelines for the U.S. domestic medical examination for newly arriving refugees. Altanta: CDC; [cited 2018 Jun 1]. Available from: http://www.cdc.gov/immigrantrefugeehealth/guidelines/domestic/domestic-guidelines.html.

[11] Benoit SR, Gregg EW, Zhou W, Painter JA. Diabetes among United States-bound adult refugees, 2009–2014. J Immigr Minor Health. 2016;18(6):1357–1364. 10.1007/s10903-016-0381-7 26976006

[12] Recommendations to prevent and control iron deficiency in the United States. Centers for Disease Control and Prevention. MMWR Recomm Rep. 1998;47:1–29. 9563847

[13] Centers for Disease Control and Prevention. Body mass index (BMI). Atlanta: CDC; [cited 2018 Jun 21]. Available from: https://www.cdc.gov/healthyweight/assessing/bmi/index.html.

[14] Centers for Disease Control and Prevention. High blood pressure fact sheet. Atlanta: CDC; [cited 2018 May 30]. Available from: https://www.cdc.gov/dhdsp/data_statistics/fact_sheets/fs_bloodpressure.htm.

[15] Mitruka K, Pezzi C, Baack B, Burke H, Cochran J, Matheson J, et al Evaluation of hepatitis B virus screening, vaccination, and linkage to care among newly arrived refugees in four states, 2009–2011. J Immigr Minor Health. 2019;21:39–46. 10.1007/s10903-018-0705-x 29417356 PMC6434685

[16] Zou G. A modified Poisson regression approach to prospective studies with binary data. Am J Epidemiol. 2004;159(7):702–6. 10.1093/aje/kwh090 15033648

[17] Trepka MJ, Pekovic V, Santana JC, Zhang G. Risk factors for lead poisoning among Cuban refugee children. Public Health Rep. 2005;120(2):179–85. 10.1177/003335490512000212 15842120 PMC1497702

[18] Markowitz G, Rosner D. "Cater to the children": the role of the lead industry in a public health tragedy, 1900–1955. Am J Public Health. 2000;90(1):36–46. 10.2105/ajph.90.1.36 10630135 PMC1446124

[19] United Nations Environment Programme. Global report on the status of legal limits on lead in paint. Nairobi: United Nations Environment Programme; 2016 5 [cited 2018 May 30]. Available from: http://wedocs.unep.org/bitstream/handle/20.500.11822/11348/Limits-Lead-Paint-2016%20Report-Final.pdf?isAllowed=y&sequence=1.

[20] Oudijk G. The rise and fall of organometallic additives in automotive gasoline. Environmental Forensics. 2010;11(1):17–49.

[21] Alvarez AM, Estevez Alvarez JR, do Nascimento CW, Gonzalez IP, Rizo OD, Carzola LL, et al Lead isotope ratios in lichen samples evaluated by ICP-ToF-MS to assess possible atmospheric pollution sources in Havana, Cuba. Environ Monit Assess. 2017;189(1):28 10.1007/s10661-016-5739-8 28000124

[22] Roberts JR, Reigart JR, Ebeling M, Hulsey TC. Time required for blood lead levels to decline in nonchelated children. J Toxicol Clin Toxicol. 2001;39(2):153–60. 10.1081/clt-100103831 11407501

[23] Morshed MG, Singh AE. Recent trends in the serologic diagnosis of syphilis. Clin Vaccine Immunol. 2015;22(2):137–47. 10.1128/CVI.00681-14 25428245 PMC4308867

[24] Centers for Disease Control and Prevention. Syphilis. Atlanta: CDC; [cited 2018 Jun 1]. Available from: https://www.cdc.gov/std/stats16/CDC_2016_STDS_Report-for508WebSep21_2017_1644.pdf.

[25] Satterwhite CL, Torrone E, Meites E, Dunne EF, Mahajan R, Ocfemia MC, et al Sexually transmitted infections among US women and men: prevalence and incidence estimates, 2008. Sex Transm Dis. 2013;40(3):187–93. 10.1097/OLQ.0b013e318286bb53 23403598

[26] World Health Organization. Proportion of children (1–14 years of age) in the country requiring preventive chemotherapy (PC) for soil-transmitted helminthiases, worldwide, 2014 Geneva: WHO; 2015 [cited 2018 Aug 22]. Available from: http://gamapserver.who.int/mapLibrary/Files/Maps/STH_2014.png?ua=1.

[27] Saboya MI, Catala L, Nicholls RS, Ault SK. Update on the mapping of prevalence and intensity of infection for soil-transmitted helminth infections in Latin America and the Caribbean: a call for action. PLoS Negl Trop Dis. 2013;7(9):e2419 10.1371/journal.pntd.0002419 24069476 PMC3777864

[28] Centers for Disease Control and Prevention. Obesity and Overweight. Atlanta: CDC; [cited 2018 May 30]. Available from: https://www.cdc.gov/nchs/fastats/obesity-overweight.htm.

[29] Centers for Disease Control and Prevention. CDC’s National Surveillance Data (2012–2016). Atlanta: CDC; [cited 2019 Feb 6]. Available from: https://www.cdc.gov/nceh/lead/data/national.htm.

[30] Centers for Disease Control and Prevention. Trends in Tuberculosis, 2016 Atlanta: CDC; [cited 2018 Jun 21]. Available from: https://www.cdc.gov/tb/publications/factsheets/statistics/tbtrends.htm.

[31] Zwerling A, Behr MA, Verma A, Brewer TF, Menzies D, Pai M. The BCG World Atlas: a database of global BCG vaccination policies and practices. PLoS Med. 2011;8(3):e1001012 10.1371/journal.pmed.1001012 21445325 PMC3062527

[32] US Department of Homeland Security. 2015 yearbook of immigration statistics. Washington: U.S. Department of Homeland Security; 2016 12 [cited 2018 Jun 22]. Available from: https://www.dhs.gov/sites/default/files/publications/Yearbook_Immigration_Statistics_2015.pdf.

