# Supplementary material for: Health screening results of Cubans settling in Texas, USA, 2010–2015: A cross-sectional analysis
Source: PLoS Med. 2020 Aug 14;17(8):e1003233. doi: 10.1371/journal.pmed.1003233 (PMC7428019; doi:10.1371/journal.pmed.1003233)
Supplement: S1 STROBE Checklist — (DOCX) [file pmed.1003233.s001.docx]

STROBE Statement—Checklist of items that should be included in reports of ***cross-sectional studies***

|  | Item No | Recommendation | Section, Paragraph # |
| --- | --- | --- | --- |
| **Title and abstract** | 1 | (*a*) Indicate the study’s design with a commonly used term in the title or the abstract | Abstract, 2 |
|  |  | (*b*) Provide in the abstract an informative and balanced summary of what was done and what was found | Abstract, 2 |
| Introduction | | | |
| Background/rationale | 2 | Explain the scientific background and rationale for the investigation being reported | Introduction, 4 |
| Objectives | 3 | State specific objectives, including any prespecified hypotheses | Introduction, 4 |
| Methods | | | |
| Study design | 4 | Present key elements of study design early in the paper | Methods, 1 |
| Setting | 5 | Describe the setting, locations, and relevant dates, including periods of recruitment, exposure, follow-up, and data collection | Methods, 1-3 |
| Participants | 6 | (*a*) Give the eligibility criteria, and the sources and methods of selection of participants | Methods, 1-3 |
| Variables | 7 | Clearly define all outcomes, exposures, predictors, potential confounders, and effect modifiers. Give diagnostic criteria, if applicable | Methods, 5-6; Table 1 |
| Data sources/ measurement | 8* | For each variable of interest, give sources of data and details of methods of assessment (measurement). Describe comparability of assessment methods if there is more than one group | Methods, 5-7; Table 1 |
| Bias | 9 | Describe any efforts to address potential sources of bias | Methods, 6-7 |
| Study size | 10 | Explain how the study size was arrived at | Methods, 1 |
| Quantitative variables | 11 | Explain how quantitative variables were handled in the analyses. If applicable, describe which groupings were chosen and why | Methods, 5; Table 1 |
| Statistical methods | 12 | (*a*) Describe all statistical methods, including those used to control for confounding | Methods, 7 |
|  |  | (*b*) Describe any methods used to examine subgroups and interactions | Methods, 7 |
|  |  | (*c*) Explain how missing data were addressed | Methods, 4 |
|  |  | (*d*) If applicable, describe analytical methods taking account of sampling strategy | n/a |
|  |  | (*e*) Describe any sensitivity analyses | Results, Table 3 |
| Results | | | |
| Participants | 13* | (a) Report numbers of individuals at each stage of study—eg numbers potentially eligible, examined for eligibility, confirmed eligible, included in the study, completing follow-up, and analysed | Results, 1 |
|  |  | (b) Give reasons for non-participation at each stage | n/a |
|  |  | (c) Consider use of a flow diagram | n/a |
| Descriptive data | 14* | (a) Give characteristics of study participants (eg demographic, clinical, social) and information on exposures and potential confounders | Results, 1-2 |
|  |  | (b) Indicate number of participants with missing data for each variable of interest | Results, 1; Table 2 |
| Outcome data | 15* | Report numbers of outcome events or summary measures | Results, 3; Table 2 |
| Main results | 16 | (*a*) Give unadjusted estimates and, if applicable, confounder-adjusted estimates and their precision (eg, 95% confidence interval). Make clear which confounders were adjusted for and why they were included | Results, 3; Table 3 |
|  |  | (*b*) Report category boundaries when continuous variables were categorized | Methods, 6; Table 1 |
|  |  | (*c*) If relevant, consider translating estimates of relative risk into absolute risk for a meaningful time period | n/a |
| Other analyses | 17 | Report other analyses done—eg analyses of subgroups and interactions, and sensitivity analyses | Results, 3; Table 3 |
| Discussion | | | |
| Key results | 18 | Summarise key results with reference to study objectives | Conclusions, 1 |
| Limitations | 19 | Discuss limitations of the study, taking into account sources of potential bias or imprecision. Discuss both direction and magnitude of any potential bias | Conclusions, 6 |
| Interpretation | 20 | Give a cautious overall interpretation of results considering objectives, limitations, multiplicity of analyses, results from similar studies, and other relevant evidence | Conclusions, 1, 7 |
| Generalisability | 21 | Discuss the generalisability (external validity) of the study results | Conclusions, 6-7 |
| Other information | | | |
| Funding | 22 | Give the source of funding and the role of the funders for the present study and, if applicable, for the original study on which the present article is based | Funding, 1 |

*Give information separately for exposed and unexposed groups.

**Note:** An Explanation and Elaboration article discusses each checklist item and gives methodological background and published examples of transparent reporting. The STROBE checklist is best used in conjunction with this article (freely available on the Web sites of PLoS Medicine at http://www.plosmedicine.org/, Annals of Internal Medicine at http://www.annals.org/, and Epidemiology at http://www.epidem.com/). Information on the STROBE Initiative is available at [www.strobe-statement.org](http://www.strobe-statement.org).

**Text excerpts:**

| **Item number** | **Description** | **Manuscript Excerpt** |
| --- | --- | --- |
| 1 | 1. Indicate the study’s design with a commonly used term in the title or the abstract 2. Provide in the abstract an informative and balanced summary of what was done and what was found | “Cubans who arrived from 2010 to 2015 and received a domestic medical examination in Texas were included. Those granted refugee/parolee status in Cuba were listed in federal databases for US-bound refugees/parolees; those were paroled after arrival were not listed. Overall, 2,189 (20%) obtained either refugee or parolee status in Cuba and 8,709 (80%) received parolee status after arrival. Approximately 62% of those who received parolee status after arrival at the border were male compared to 49% of those who obtained prior refugee/parolee status in Cuba. Approximately half (45%) of those paroled after arrival were 19-34 years old (vs. 26% among those who obtained refugee/parolee status in Cuba). Separate models were created for each screening indicator as the outcome, with entry route as the main exposure variable. Crude and adjusted prevalence ratios were estimated using PROC GENMOD procedures in SAS 9.4. Individuals paroled after arrival were less likely to screen positive for parasitic infections (9.6% vs. 12.2%; adjusted prevalence ratio: 0.79, 0.71-0.88) and elevated blood lead levels (children ≤16 years old, 5.2% vs. 12.3%; adjusted prevalence ratio: 0.42, 0.28-0.63).” |
| 2 | Explain the scientific background and rationale for the investigation being reported. | “Much is known about disease prevalence among other populations resettling in the US, but the health status of Cubans has not been previously examined. Additionally, little is known regarding the differences in health status upon arrival between individuals paroled at the border and individuals who obtained refugee/parolee status in Cuba.” |
| 3 | State specific objectives, including any prespecified hypotheses | “We hypothesized that, because these two groups differed by entry route, and likely life experiences, we might also observe differences in health status.”  “We compared health records from initial post-arrival domestic medical examinations of Cubans entering Texas to identify health differences that can inform targeted screening and long-term health management strategies.” |
| 4 | Present key elements of study design early in the paper | “We conducted a retrospective cross-sectional analysis of the Texas Department of State Health Services (DSHS) database examining differences in health status at the voluntary initial domestic (after US arrival) medical examinations among Cubans paroled at the border and those who obtained refugee/parolee status in Cuba. Cuban refugees and parolees who arrived in Texas between January 1, 2010, and September 30, 2015, and who received a domestic medical examination, were included.” |
| 5 | Describe the setting, locations, and relevant dates, including periods of recruitment, exposure, follow-up, and data collection | “Cuban refugees and parolees who arrived in Texas between January 1, 2010, and September 30, 2015, and who received a domestic medical examination, were included.”  “The Refugee Health Program within DSHS collected health screening and demographic data from all seven of the public health departments that perform domestic medical examinations for refugees and parolees. Data were retrieved from the state’s database while the program was under DSHS (in 2016 the program moved to the US Committee for Refugees and Immigrants).”    “DSHS data were cross-referenced with EDN and WRAPS to determine entry route [8-9]. Individuals who obtained refugee/parolee status in Cuba were identified through the presence of a record in EDN and/or WRAPS, indicating they received a pre-departure overseas examination and an approval for refugee/parolee status before entry. Those who were paroled at the border were defined as individuals not listed in EDN or WRAPS.” |
| 6 | (*a*) Give the eligibility criteria, and the sources and methods of selection of participants | “Cuban refugees and parolees who arrived in Texas between January 1, 2010, and September 30, 2015, and who received a domestic medical examination, were included.”  “The Refugee Health Program within DSHS collected health screening and demographic data from all seven of the public health departments that perform domestic medical examinations for refugees and parolees. Data were retrieved from the state’s database while the program was under DSHS (in 2016 the program moved to the US Committee for Refugees and Immigrants).”    “DSHS data were cross-referenced with EDN and WRAPS to determine entry route [8-9]. Individuals who obtained refugee/parolee status in Cuba were identified through the presence of a record in EDN and/or WRAPS, indicating they received a pre-departure overseas examination and an approval for refugee/parolee status before entry. Those who were paroled at the border were defined as individuals not listed in EDN or WRAPS.” |
| 7 | Clearly define all outcomes, exposures, predictors, potential confounders, and effect modifiers. Give diagnostic criteria, if applicable | “Demographic data and health variables collected during the domestic medical examination included sex; age; body mass index (BMI; calculated using weight and height); blood pressure; hemoglobin and hematocrit results (to identify potential for anemia); blood lead levels (BLL); laboratory screening results for hepatitis B (serologic testing of hepatitis B surface antigen, hepatitis B surface antibody, and total hepatitis B core antibody), hepatitis C (antibody test), and HIV infection, eosinophilia, and screening for Mycobacterium tuberculosis infection by tuberculin skin test (TST) and/or interferon gamma release assay (IGRA). For syphilis, all clinics screened using rapid plasma reagin (RPR) followed by Treponema pallidum particle agglutination (TPPA) assay (three clinics also initially screened with IgG). Two clinics reported only RPR results to DSHS; all others reported results following the full sequence. Clinical results were unavailable to distinguish latent from active infection. Entrants were also screened for parasitic infections (parasites assessed at the state laboratory included ascaris, clonorchis, dientamoeba, amoebas, giardia, hookworm, and trichuris by ova and parasite examination using a two stool sample per individual; strongyloides and schistosoma by serology with an enzyme immunoassay). For all health variables, only screening results are reported. Verified clinical diagnosis data were unavailable. Table 1 provides classifications and interpretations for each health assessment component (additional information on methods for outcome measurement or cutoff values aside from that presented in the text and Table 1 were unavailable).”  “Table 1. Classification of health assessment components reported at Texas domestic medical screening examination of Cuban entrants, 2010-2015”  “Because only one blood pressure reading was available (clinical guidance suggests ≥2 separate readings to diagnose), a hypertension diagnosis was not recorded; rather, individuals were classified as having “normal” or “elevated” blood pressure. Similarly, only “potential for anemia” was recorded, because additional factors (pregnancy, menstruation, repeat testing) often accounted for in official diagnoses were unavailable [11]. BMI and blood pressure were analyzed for individuals ≥18 years old, and BLL for children ≤16 years, in accordance with CDC guidelines [10, 12-13]. Of those screened for syphilis, 98% were ≥15 years old as recommended by CDC [10].” |
| 8 | For each variable of interest, give sources of data and details of methods of assessment (measurement). Describe comparability of assessment methods if there is more than one group | “Demographic data and health variables collected during the domestic medical examination included sex; age; body mass index (BMI; calculated using weight and height); blood pressure; hemoglobin and hematocrit results (to identify potential for anemia); blood lead levels (BLL); laboratory screening results for hepatitis B (serologic testing of hepatitis B surface antigen, hepatitis B surface antibody, and total hepatitis B core antibody), hepatitis C (antibody test), and HIV infection, eosinophilia, and screening for Mycobacterium tuberculosis infection by tuberculin skin test (TST) and/or interferon gamma release assay (IGRA). For syphilis, all clinics screened using rapid plasma reagin (RPR) followed by Treponema pallidum particle agglutination (TPPA) assay (three clinics also initially screened with IgG). Two clinics reported only RPR results to DSHS; all others reported results following the full sequence. Clinical results were unavailable to distinguish latent from active infection. Entrants were also screened for parasitic infections (parasites assessed at the state laboratory included ascaris, clonorchis, dientamoeba, amoebas, giardia, hookworm, and trichuris by ova and parasite examination using a two stool sample per individual; strongyloides and schistosoma by serology with an enzyme immunoassay). For all health variables, only screening results are reported. Verified clinical diagnosis data were unavailable. Table 1 provides classifications and interpretations for each health assessment component (additional information on methods for outcome measurement or cutoff values aside from that presented in the text and Table 1 were unavailable).”  “Table 1. Classification of health assessment components reported at Texas domestic medical screening examination of Cuban entrants, 2010-2015”  “Because only one blood pressure reading was available (clinical guidance suggests ≥2 separate readings to diagnose), a hypertension diagnosis was not recorded; rather, individuals were classified as having “normal” or “elevated” blood pressure. Similarly, only “potential for anemia” was recorded, because additional factors (pregnancy, menstruation, repeat testing) often accounted for in official diagnoses were unavailable [11]. BMI and blood pressure were analyzed for individuals ≥18 years old, and BLL for children ≤16 years, in accordance with CDC guidelines [10, 12-13]. Of those screened for syphilis, 98% were ≥15 years old as recommended by CDC [10].”  “Chi-square comparisons were conducted to examine differences between the two entry routes (α < 0.05).” |
| 9 | Describe any efforts to address potential sources of bias | “Because only one blood pressure reading was available (clinical guidance suggests ≥2 separate readings to diagnose), a hypertension diagnosis was not recorded; rather, individuals were classified as having “normal” or “elevated” blood pressure. Similarly, only “potential for anemia” was recorded, because additional factors (pregnancy, menstruation, repeat testing) often accounted for in official diagnoses were unavailable [11].”  “Crude and adjusted prevalence ratios (PR), controlling for the county of medical examination (proxy for clinic) to limit bias due to possible differences in screening processes, were estimated using PROC GENMOD procedures (modified Poisson regression approach that ensures robust and conservative error estimation [14]; log-link function) in SAS 9.4 (Cary, NC, USA). Confounding by sex and age, and two-way interactions by age (categorical variable; age as a continuous variable did not meet the linear assumption required for this type of model) with entry route and sex with entry route for each model were assessed to determine the most parsimonious model. Interaction terms were retained if significant (p<0.05) and confounding terms were eliminated if the estimate did not differ by more than 10% without the inclusion of the potential confounder.” |
| 10 | Explain how the study size was arrived at | “Cuban refugees and parolees who arrived in Texas between January 1, 2010, and September 30, 2015, and who received a domestic medical examination, were included.” |
| 11 | Explain how quantitative variables were handled in the analyses. If applicable, describe which groupings were chosen and why | “Table 1 provides classifications and interpretations for each health assessment component (additional information on methods for outcome measurement or cutoff values aside from that presented in the text and Table 1 were unavailable).” |
| 12 | (*a*) Describe all statistical methods, including those used to control for confounding  (*b*) Describe any methods used to examine subgroups and interactions  (*c*) Explain how missing data were addressed  (*e*) Describe any sensitivity analyses | “Crude and adjusted prevalence ratios (PR), controlling for the county of medical examination (proxy for clinic) to limit bias due to possible differences in screening processes, were estimated using PROC GENMOD procedures (modified Poisson regression approach that ensures robust and conservative error estimation [14]; log-link function) in SAS 9.4 (Cary, NC, USA). Confounding by sex and age, and two-way interactions by age (categorical variable; age as a continuous variable did not meet the linear assumption required for this type of model) with entry route and sex with entry route for each model were assessed to determine the most parsimonious model. Interaction terms were retained if significant (p<0.05) and confounding terms were eliminated if the estimate did not differ by more than 10% without the inclusion of the potential confounder”  “Of note, all Cuban refugees/parolees listed in EDN as resettling in Texas were found in our dataset (i.e., completed domestic medical examination), indicating there were no missing individuals from our denominator among those who obtained status in Cuba.”  Table 2 footnote: “Where percentages do not add to 100%, values were missing in the data set”  Table 3 footnote: “Sensitivity analysis that categorizes potential anemia as abnormal hemoglobin or abnormal hematocrit: 16% within anemia in total population (15% among those paroled at the border, 19% among those who obtained status in Cuba); crude PR: 0.80 (0.75-0.86); adjusted PR: 0.94 (0.87-1.01)” |
| 13 | (a) Report numbers of individuals at each stage of study—eg numbers potentially eligible, examined for eligibility, confirmed eligible, included in the study, completing follow-up, and analysed | “A total of 10,898 Cubans were included. Of those, 8,709 (80%) were paroled at the border. The remaining 2,189 (20%) obtained refugee/parolee status in Cuba.” |
| 14 | 1. Give characteristics of study participants (eg demographic, clinical, social) and information on exposures and potential confounders 2. Indicate number of participants with missing data for each variable of interest | “Of those included, 41% were female, and average age was 33.5 years (SD: 14.1; 13% ≤18 years old) (Table 2). Approximately 66% were overweight or had obesity (33% normal BMI, 1% underweight), and over half (56%) had elevated blood pressure. Four percent had a positive TST and/or IGRA, indicating further testing was required to determine tuberculosis disease (≤18 years old: 1.2%). Less than 1% of those tested screened positive for hepatitis B, and over half (57%) were classified as susceptible (as defined in Table 1). Ten percent screened positive for at least one parasite. One hundred and thirty individuals screened positive for syphilis (1.2%; all ≥18 years old), of which 92% were identified in clinics that submitted results after the full RPR/TPPA sequence. Approximately 4% had both abnormal hematocrit and hemoglobin results suggesting potential anemia (16% abnormal hemoglobin only, 4% abnormal hematocrit only). Of the 1,178 children with valid BLL results, 8% recorded a level higher than CDC’s reference level of ≥5 ug/dL (0.8% ≥10 ug/dL).”  “Approximately 62% of those paroled at the border were male compared to 49% who obtained status in Cuba. Approximately half (45%) of those paroled at the border were 19–34 years old (vs. 26% among those who received status in Cuba). Chi-square tests revealed significant differences in gender, age, BMI, hepatitis B infection and susceptibility, HIV infection, parasitic infection(s), presence of eosinophilia, potential anemia, and elevated blood lead levels (EBBL) between the two entry routes (Table 2).”  Table 2 footnote: “Where percentages do not add to 100%, values were missing in the data set” |
| 15 | Report numbers of outcome events or summary measures | “Females paroled at the border were more likely than females who received status in Cuba to be overweight or have obesity [adjPR: 1.06 (1.02–1.11); controlling for age]. This correlation was the opposite for males [adjPR: 0.88 (0.85–0.92)], controlling for age. For both sexes, those paroled at the border were less likely to be infected with at least one parasite [adjPR: 0.79 (0.71–0.88)], but more likely to have eosinophilia [females adjPR: 2.00 (1.80–2.23), males adjPR: 1.48 (1.26–1.73)]. Males paroled at the border were more likely to have a positive syphilis screening [adjPR: 1.24 (1.06–1.46)], whereas their female counterparts were less likely [adjPR: 0.32 (0.12–0.80)]. Among the 1,178 children ≤16 years old, those paroled in at the border were less likely to have EBLL of ≥5 ug/dL [adjPR: 0.42 (0.28–0.63)].”  Table 2. Demographic characteristics and clinical screening results of individuals from Cuba resettling in Texas 2010-2015, by route of entry (N, %) |
| 16 | *(a)* Give unadjusted estimates and, if applicable, confounder-adjusted estimates and their precision (eg, 95% confidence interval). Make clear which confounders were adjusted for and why they were included  (*b*) Report category boundaries when continuous variables were categorized | “Females paroled at the border were more likely than females who received status in Cuba to be overweight or have obesity [adjPR: 1.06 (1.02–1.11); controlling for age]. This correlation was the opposite for males [adjPR: 0.88 (0.85–0.92)], controlling for age. For both sexes, those paroled at the border were less likely to be infected with at least one parasite [adjPR: 0.79 (0.71–0.88)], but more likely to have eosinophilia [females adjPR: 2.00 (1.80–2.23), males adjPR: 1.48 (1.26–1.73)]. Males paroled at the border were more likely to have a positive syphilis screening [adjPR: 1.24 (1.06–1.46)], whereas their female counterparts were less likely [adjPR: 0.32 (0.12–0.80)]. Among the 1,178 children ≤16 years old, those paroled in at the border were less likely to have EBLL of ≥5 ug/dL [adjPR: 0.42 (0.28–0.63)].”  Table 3. Crude and adjusted prevalence ratios (PR) of domestic medical screening status, Cubans in Texas who were paroled into the United States at the border vs. those who obtained refugee/Cuban entrant status in Cuba (reference), 2010-2015  Table 1. Classification of health assessment components reported at Texas domestic medical screening examination of Cuban entrants, 2010-2015 |
| 17 | Report other analyses done—eg analyses of subgroups and interactions, and sensitivity analyses | “Females paroled at the border were more likely than females who received status in Cuba to be overweight or have obesity [adjPR: 1.06 (1.02–1.11); controlling for age]. This correlation was the opposite for males [adjPR: 0.88 (0.85–0.92)], controlling for age. For both sexes, those paroled at the border were less likely to be infected with at least one parasite [adjPR: 0.79 (0.71–0.88)], but more likely to have eosinophilia [females adjPR: 2.00 (1.80–2.23), males adjPR: 1.48 (1.26–1.73)]. Males paroled at the border were more likely to have a positive syphilis screening [adjPR: 1.24 (1.06–1.46)], whereas their female counterparts were less likely [adjPR: 0.32 (0.12–0.80)]. Among the 1,178 children ≤16 years old, those paroled in at the border were less likely to have EBLL of ≥5 ug/dL [adjPR: 0.42 (0.28–0.63)].”  Table 3 footnote: “Sensitivity analysis that categorizes potential anemia as abnormal hemoglobin or abnormal hematocrit: 16% within anemia in total population (15% among those paroled at the border, 19% among those who obtained status in Cuba); crude PR: 0.80 (0.75-0.86); adjusted PR: 0.94 (0.87-1.01)” |
| 18 | Summarise key results with reference to study objectives | “Overall, our analysis suggests that the health profiles of individuals paroled into the United States at the border and those who obtained refugee/parolee status in Cuba during 2010-2015 may differ with respect to certain health outcomes (e.g., blood lead levels in children). However, for some health outcomes, our results are inconclusive.” |
| 19 | Discuss limitations of the study, taking into account sources of potential bias or imprecision. | “Our analysis was subject to several limitations. Only screening results were used, indicating potential for misclassification and/or prevalence overestimation/underestimation in the event of indeterminate or false positive/negative results. Secondly, only 2010-2015 Texas data was used, and therefore, the results may not be representative of Cubans who resettled outside of Texas or in recent years. Entry route misclassification also cannot be ruled out (e.g., individual received an overseas medical examination but chose to cross the border). Although suspected to be small it is unclear what proportion could have been misclassified in this manner. Only those who received refugee/parolee status in Cuba received an overseas medical exam, however, because this exam only screens for inadmissible conditions, the impact would only occur in the syphilis and tuberculosis models. Nonetheless, our ultimate goal is to understand health differences between the two groups upon arrival to the US to appropriately treat and care for them after arrival. Therefore, although we cannot control for the medical care prior to US arrival, we do not believe this to be of major issue in terms of our overall message. Additionally, the data set is likely missing individuals paroled at the border who did not receive a domestic medical screening. This omission potentially introduced bias if these individuals were demographically or clinically different from those examined; however, data are not available to asses if differences exist. Lack of access to clinic-level information prevented the use of laboratory-specific cutoff values for abnormal results, and missing information limited our ability to make complex inferences (e.g., information about pregnancy, which impacts hemoglobin/hematocrit results interpretation, and history of Bacille Calmette-Guerin vaccine, which may cause a false positive TST reaction, were unavailable) [30]. Additionally, without vaccination history, hepatitis B serologic testing interpretation is difficult because, for some, hepatitis B surface antibody wanes post-vaccination, yet protection persists through immune memory. In other cases, differentiation between susceptibility and low-level hepatitis B infection using only serology results is difficult. Although nine of the ten models included >80% of the study population, missing data limited our interpretations, particularly for hepatitis C. Lack of data availability on socioeconomic status or education level also prevented the ability to adjust our models based on these factors. Ultimately, our estimates are merely a cross-sectional view and cannot identify a directional relationship, nor account for differences related to travel time or geographic route. Finally, given the large sample size, even minimal differences were significant. Yet differences in lead levels, parasitic infections/eosinophilia, and syphilis have clinical and public health significance, and should continue to be investigated.” |
| 20 | Give a cautious overall interpretation of results considering objectives, limitations, multiplicity of analyses, results from similar studies, and other relevant evidence | “The mechanisms for the patterns we observed remain unclear but may include differences in exposures both en route and before departure (potentially related to socioeconomic status or education), duration of the journey from Cuba to the US that potentially impacted presentation or resolution of the health condition, and receipt of overseas medical examination and treatments provided. Additionally, human migration is complex and influenced by a variety of geopolitical and socioeconomic factors. Therefore, it is also possible the differences observed occurred by chance or were driven by self-selection of route.”  “Although the Cuban Haitian Entrant Program’s policies changed in 2017 regarding those paroled at the border [5], thousands of Cubans entered under its premises [2, 6, 31]. As outlined, among Cubans residing in the United States there exist two distinct subpopulations that differ in not only life experiences and entry routes but also health profiles.” |
| 21 | Discuss the generalisability (external validity) of the study results | “Secondly, only 2010-2015 Texas data was used, and therefore, the results may not be representative of Cubans who resettled outside of Texas or in recent years.”  “Although the Cuban Haitian Entrant Program’s policies changed in 2017 regarding those paroled at the border [5], thousands of Cubans entered under its premises [2, 6, 31]. As outlined, among Cubans residing in the United States there exist two distinct subpopulations that differ in not only life experiences and entry routes but also health profiles. Therefore, understanding the health status of these two groups can be used to inform US-based public health recommendations and develop intervention strategies targeted to each subpopulation.” |
| 22 | Give the source of funding and the role of the funders for the present study and, if applicable, for the original study on which the present article is based | “Funding: This analysis was supported in part by an appointment (ES) to the Applied Epidemiology Fellowship Program administered by the Council of State and Territorial Epidemiologists (CSTE) and funded by the Centers for Disease Control and Prevention (CDC) Cooperative Agreement Number 1U38OT000143-05. The funders had no role in study design, data collection and analysis, decision to publish, or preparation of the manuscript.” |
